# Leptin’s metabolic and immune functions can be uncoupled at the ligand/receptor interaction level

**DOI:** 10.1007/s00018-014-1697-x

**Published:** 2014-08-07

**Authors:** Lennart Zabeau, Cathy J. Jensen, Sylvie Seeuws, Koen Venken, Annick Verhee, Dominiek Catteeuw, Geert van Loo, Hui Chen, Ken Walder, Jacob Hollis, Simon Foote, Margaret J. Morris, José Van der Heyden, Frank Peelman, Brian J. Oldfield, Justin P. Rubio, Dirk Elewaut, Jan Tavernier

**Affiliations:** 1 Department of Medical Protein Research, Faculty of Medicine and Health Sciences, Flanders Institute for Biotechnology, Ghent University, A. Baertsoenkaai 3, 9000 Ghent, Belgium; 2 Neurogenetics Laboratory, Howard Florey Institute, Melbourne, Australia; 3 Laboratory for Molecular Immunology and Inflammation, Department of Rheumatology, Ghent University Hospital, Ghent University, De Pintelaan 185, 9000 Ghent, Belgium; 4 Inflammation Research Center, Unit of Molecular Signal Transduction in Inflammation, Faculty of Sciences, Flanders Institute for Biotechnology, Ghent University, Ghent, Belgium; 5 Department of Pharmacology, University of Melbourne, Melbourne, Australia; 6 Metabolic Research Unit, School of Medicine, Deakin University, Geelong, Australia; 7 Department of Physiology, Monash University, Melbourne, Australia; 8 Menzies Research Institute, Hobart, Australia

**Keywords:** Leptin receptor, Antagonist, Genetic model, Nanobody, Obesity, Metabolism, Autoimmune disease

## Abstract

The adipocyte-derived cytokine leptin acts as a metabolic switch, connecting the body’s metabolism to high-energy consuming processes such as reproduction and immune responses. We here provide genetic and biochemical evidence that the metabolic and immune functions of leptin can be uncoupled at the receptor level. First, homozygous mutant *fatt/fatt* mice carry a spontaneous splice mutation causing deletion of the leptin receptor (LR) immunoglobulin-like domain (IGD) in all LR isoforms. These mice are hyperphagic and morbidly obese, but display only minimal changes in size and cellularity of the thymus, and cellular immune responses are unaffected. These animals also displayed liver damage in response to concavalin A comparable to wild-type and heterozygous littermates. Second, treatment of healthy mice with a neutralizing nanobody targeting IGD induced weight gain and hyperinsulinaemia, but completely failed to block development of experimentally induced autoimmune diseases. These data indicate that leptin receptor deficiency or antagonism profoundly affects metabolism, with little concomitant effects on immune functions.

**Electronic supplementary material:**

The online version of this article (doi:10.1007/s00018-014-1697-x) contains supplementary material, which is available to authorized users.

## Introduction

Leptin, the 16 kDa adipocyte hormone product of the *ob* gene, influences a multitude of biological processes, including immunity [1], reproduction [2], linear growth [3], glucose homeostasis [4] and bone metabolism [5]. However, it is best known for its dramatic effect as a satiety signal, since mouse strains deficient in leptin signalling are hyperphagic and obese [6]. Primarily produced in adipocytes, leptin provides information about the availability of energy stores and functions predominantly, but not exclusively, at the level of the mediobasal hypothalamus to modulate feeding and energy expenditure, thus regulating body weight. Adequate leptin signalling appears to be permissive for energy expensive processes such as linear growth, reproduction and adequate immune responses, all of which are dysregulated when the leptin signalling pathways are compromised.

Leptin plays a role in both innate and adaptive immunity (reviewed in [1]) and leptin deficiency causes immune dysfunction and increased risk of infection in mice and man [7, 8]. In innate immunity, it promotes secretion of inflammatory cytokines and the activation of macrophages, neutrophils and natural killer cells. Functions in adaptive immunity include thymic homeostasis, naïve CD4^+^ cell proliferation and promotion of T helper 1 (T_H_1) responses. In addition, leptin suppresses the expansion of CD4^+^CD25^high^ regulatory T cells (T_Regs_) that dampen immune reactions [9]. Leptin’s role in CD4^+^ T cell-mediated responses links the hormone to the onset and progression of several T cell-controlled autoimmune diseases, including Crohn’s disease [10], rheumatoid arthritis [11], multiple sclerosis [12, 13] and autoimmune hepatitis [14–16].

Six LR isoforms (LRa-f) with an identical extracellular domain are produced by alternative splicing or ectodomain shedding: one long, four short and one extracellular soluble variant. The LR long form (LRlo or LRb) is the only variant capable of efficient signalling and is highly expressed in certain nuclei of the hypothalamus [17], a region of the brain involved in the regulation of body weight. A 106 nucleotide insertion precisely at the junction where the long and short form transcripts diverge in the *lr* gene results in premature termination of the LRlo intracellular domain and concomitant loss of hypothalamic signalling, thus explaining the obese phenotype of *db/db* mice [18]. Functional LRlo expression is also observed in several peripheral cell types, including cells of the immune system [17]. In line with this, PET imaging revealed significant leptin binding to immune and hematopoietic cell types [19].

LR is a member of the class I cytokine receptor family [20]. Its ectodomain is composed of two cytokine receptor homology (CRH1 and CRH2) domains, which are separated by an immunoglobulin-like domain (IGD) and followed by two membrane-proximal fibronectin type III (FN III) domains. The CRH2 domain is necessary and sufficient for leptin binding [21, 22], but receptor clustering requires interaction with IGD as leptin mutants that fail to contact this domain behave as leptin antagonists [23]. Like all class I cytokine receptors, the LR lacks intrinsic kinase activity and relies for signalling on constitutively associated JAK2, a member of the Janus tyrosine kinase family [24]. LR clustering results in JAK2 transphosphorylation and activation of several intracellular signalling cascades including the STAT, MAPK, PI3 K and mTOR pathways (reviewed in [25]).

In this study, we provide the first genetic and biochemical evidence that different leptin-driven biological processes can be uncoupled at the ligand/receptor interaction level.

## Materials and methods

### Reagents

Mouse leptin was produced and purified as described earlier [26] and 4.10-mAlb by the VIB Protein Service Facility up to 95 % purity. LPS contaminations were less than 1 EU per mg protein. LPS content was measured using the limulus amebocyte lysate in combination with a chromogenic substrate (Cambrex), or with the Toll-like receptor 4 expressing Hek293-BlueTM cells (InvivoGen) according to the manufacturer’s instructions. Antibodies Alexafluor labelled anti-CD4 and PE labelled anti-CD8 (both from eBiosciences) were used according to the manufacturer’s instructions.

### Animals

FATT experiments: Mice used were of the 129/SvEvTac strain and kindly provided by Dr Nancy Jenkins at the National Cancer Institute-Frederick, Maryland, USA. The obese phenotype arose during establishment of the colony at the Walter and Eliza Hall Institute, Melbourne, Australia. Mice had ad libitum access to standard lab chow and water and were on a 12-h light–dark cycle with lights-on at 07:00. Approval for the work described was granted by the Howard Florey Institute Animal Ethics Committee (AEC) and Monash University School of Biological Sciences AEC. Mice of approximately 10 weeks of age were used for the phenotypic characterization. For further experiments (thymus and spleen characterization and the Con A experiments), mice were backcrossed onto the C57BL/6 genetic background for at least 8 generations. Nanobody experiments: C57BL/6 and DBA/1 mice were purchased from Harlan Netherlands and Janvier, respectively. Animals were treated and used in agreement with the institutional guidelines.

### Sequencing and transcript analysis

Genomic DNA was isolated from tail tips using Proteinase K (Sigma) with isopropanol purification. Hypothalamic tissue was dissected from mouse brains, and RNA was extracted using the RNeasy lipid tissue Mini-kit™ (Qiagen). Reverse transcription was performed on 200 ng mRNA using Superscript III™ (Invitrogen). Oligonucleotide primers for PCR and direct sequencing were designed using the Primer 3 program and PCR was performed under standard conditions using TaqGold (Roche). PCR products were purified using Magnesil Paramagnetic Particles (Promega), and DNA sequencing was performed on an ABI 377 DNA sequencing machine using Big Dye terminator chemistry.

### Phenotypic studies

Blood was collected between 09:00 and 11:00 by retro-orbital bleed from conscious, non-fasting animals. Glucose was tested immediately on whole blood using an “Accu-Chek Advantage” glucometer. Tissues were taken from animals killed with an ip lethal injection of pentobarbital sodium (Lethobarb, 100 mg/kg, Virbac). Mice were weighed and measured from snout to anus along the ventral surface before undergoing dissection and tissue collection. Organs and fat pads were collected and weighed.

### ConA induced hepatitis

Hepatitis was induced by an intravenous injection of indicated amounts Con A (Applichem) dissolved in pyrogen-free PBS. At different times after injection, blood was collected and serum prepared. Serum alanine aminotransferase (ALT) and aspartate aminotransferase (AST) levels were measured using a standard photometric method.

### Collagen-induced arthritis (CIA)

Chicken type-II (CII) collagen (MB Biosciences) was dissolved in 0.1 M acetic acid and emulsified with complete Freund’s adjuvant (CFA) (incomplete Freund’s adjuvant (sigma) + *Mycobacterium tuberculosis* (Difco)). 100 µl of this emulsion was injected subcutaneously at the base of the tail of DBA/1 mice. Twenty-one days later, the animals were challenged with a second injection of CII emulsified in incomplete Freund’s adjuvant. Mice were treated ip with 4.10-mAlb (100 µg/mouse/day) or PBS starting at day 20. Clinical severity of arthritis was graded as follows: 0: normal paws; 0.5: erythema and edema of one digit; 1: erythema and slight edema of the tarsus or one joint; 2: erythema and moderate edema of the tarsus or more than 1 joint; 3: erythema and severe edema involving the entire paw; 4: ankylosis and deformation. Scoring was performed by an investigator unaware of the mouse identity. The arthritic score for each mouse was obtained as the sum of the score recorded for each limb individually.

### Induction and assessment of experimental autoimmune encephalomyelitis (EAE)

Male mice, 10–15 weeks of age, received subcutaneous injection of 200 μg MOG_35–55_ peptide (Sigma) in 200 μl sterile PBS emulsified with an equal volume of complete Freund’s adjuvant (Sigma) containing 5 mg/ml Mycobacterium tuberculosis H37Ra (BD Biosciences). Mice also received ip 50 ng pertussis toxin (Sigma) in 200 μl sterile PBS, at the time of immunization and 48 h later. Starting at day 7, mice were injected daily with PBS of 4.10-mAlb (100 μg/mouse/day). Animals were weighed and clinically scored blindly on a daily basis: Clinical signs of disease were scored as follows: 0: normal; 1: weakness of the tail; 2: complete loss of tail tonicity; 3: partial hind limb paralysis; 4: complete hind limb paralysis; 5: forelimb paralysis or moribund; 6: death. 0.5 points were added for immediate clinical findings.

### Thymocytes and splenocytes cultures

Mice were killed by cervical dislocation and organs dissected. Lymphocytes were isolated by mincing and passing through a nylon cell strainer (BD Falcon). Red blood cells were lysed for 15 min in ACK (ammonium–chloride–potassium) lysis buffer. Cell subpopulations were counted and analysed by 2-colour flow cytometry using a FACScalibur (Becton–Dickinson) with the appropriate FITC- or PE-conjugated mAbs (see above).

Following splenocyte isolation, 6 × 10^5^ cells were cultured in 200 µl DMEM supplemented with 10 % FCS, 1 % glutamine and 1 % Penicillin/Streptamycin in de presence of Con A (as indicated). Cells were plated into 96-well culture plates (Cellstar, Greiner Bio-One) and after 96 h of culture, supernatant was collected for cytokine detection.

### Cytokine and SOL LR profiling

Serum COMP (AnaMar Medical), mouse IL-18 (MBL), mouse insulin (Mercodia), mouse IFN-γ, mouse TNF-α, mouse IL-4 (all R&D systems) and (total) mouse leptin (R&D Systems) were determined using ELISA’s according to the manufacturer’s instructions. Free leptin levels were quantified by coating Maxisorp plates (Costar) overnight with mLR_EC_ purified protein (2 μg/ml in coating buffer (50 mM NaCO_3_; pH 10,6)). After blocking, plates were incubated with serum of treated mice. Leptin was detected with a polyclonal secondary anti-leptin Ab and streptavidin-HRP (R&D Systems).

SOL LR levels were determined as follows: a 1,000-fold dilution of mouse serum, or a serial dilution purified mLR_EC_ as a standard, was allowed to bind to penta-His Ab (Qiagen) coated Maxisorp plates (Costar). After washing, plates were incubated for 2 h at room temperature with a 1/50 dilution of a COS-1 conditioned medium containing the leptin-SEAP chimera (final concentration ±10 ng/ml). Bound secreted alkaline phosphatase activity was measured using the chemiluminescent CSPD substrate (PhosphaLight, Tropix) in a TopCount chemiluminescence counter.

### Construction of LR-FATT1

The expression plasmid pMET7 LR-FATT1 was constructed by introducing a Nhe I site just before and just after the Ig-like domain coding sequence in the pMET7 LR plasmid using site-directed mutagenesis (Stratagene). The resulting vector was Nhe I cut and circularized.

### Cell lines and transfection procedures

Hek293T, Hek293-BlueTM cells (InvivoGen) and MCF7 cells were cultured in 10 % CO_2_ humidified atmosphere at 37 °C and grown using DMEM with 4,500 mg/l glucose, 10 % foetal bovine serum and 50 μg/ml gentamicin (all from Invitrogen). Hek293T cells were transfected using a standard calcium phosphate precipitation procedure [22].

### Reporter assay, leptin binding assay and western blot analysis

Reporter assay: Hek293T cells were transiently co-transfected with the appropriate receptors and the STAT3 responsive pXP2d2-rPAP1 (rat pancreatitis associated protein 1)-luciferase reporter [27]. Transfected cells were stimulated overnight as indicated. Luciferase activity was measured for 5 s in a TopCount chemiluminescence counter (Packard).

Leptin binding properties of LR variants: leptin binding was measured in a binding assay with a mouse leptin-SEAP chimeric protein as described earlier [23]. In brief, transfected Hek293T cells were incubated with a 1/50 dilution leptin-SEAP conditioned medium (see above). Cells were washed and bound secreted alkaline phosphatase activity was measured using the chemiluminescent CSPD substrate (PhosphaLight, Tropix) in a TopCount chemiluminescence counter.

*Western blot* For STAT3 and JAK2 phosphorylation, transfected Hek293T cells were serum-starved overnight and stimulated for 15 min with ligand. Phosphorylated and total protein levels were detected with, respectively, anti-phospho STAT3 (Tyr705), anti-STAT3 (both from Cell Signalling), or anti-phospho JAK2 (Tyr 1007/1008), anti-JAK2 (both from Upstate Biotechnology) using standard western blot techniques. To detect JAK2 phosphorylation and expression, 0.01 μg pRK5-JAK2 was co-transfected.

### Statistical analysis

Anatomical and blood measurements were analysed based on genotype and sex. Statistical analyses were conducted using two-way ANOVA in SPSS 15 (SPSS, Australia). When significant effects or interactions were evident, post hoc comparisons were performed using Tukey’s HSD test when Levene’s test for homogeneity of variance was satisfied and Games–Howell test when it was not. For analyses of two groups, Mann–Whitney *U* tests were performed. *P < 0.05 and **P < 0.001. SPSS and GraphPad software was used for statistical data analysis.

## Results

### Obese *fatt/fatt mice* carry a leptin receptor lacking the IGD

**Fig. 1:**
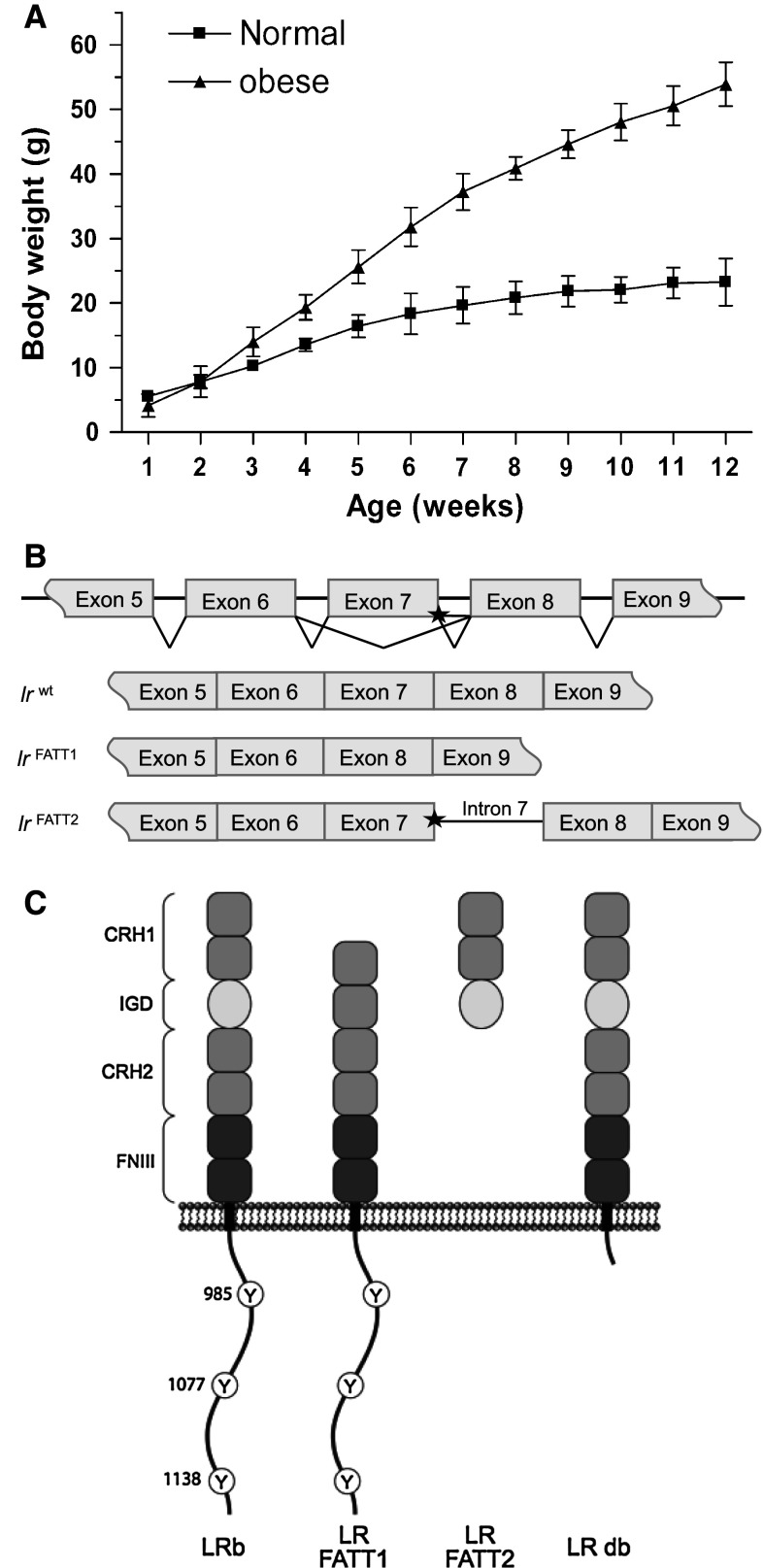
Properties and effect of the FATT mutation in vivo. **a** Weekly body weights of male mice in “C” generation (grandparental offspring of a cross between A1 and A3). Results are shown as the mean (±STDEV). Mice were grouped as “normal” or obese at 12 weeks of age (normal *n* = 5, obese *n* = 4). **b** LR and LR-FATT alternatively spliced transcripts. Sequence differences suggest the existence of two alternatively spliced transcripts: LR-FATT1 (a 291-bp deletion corresponding to the entire coding region of exon 7) and LR-FATT2 (retains 152 bp of intron 7 sequence between exons 7 and 8). **c** Putative LR-FATT proteins compared to wild-type and LR db receptors. The wild-type full-length receptor (LRb) contains all described domains of the leptin receptor. The IGD main is excised from LR-FATT1, while LR-FATT2 is truncated just after this domain. The LR db receptor is truncated after box 1. *CRH* cytokine receptor homology, *IGD* immunoglobulin-like, *FN III* fibronectin type III, *Y* tyrosine

The obese *fatt/fatt* phenotype was first noticed when a rapid post-weaning weight gain was observed in a proportion (12 out of 58 animals) of progeny arising from two independent brother–sister matings of the same sibship in 129/SvEvTac mice. By 25 weeks of age, obese *fatt/fatt* mice, without any sex bias, generally reached 75 g, approximately three times the average adult weight of wild-type animals (Fig. 1a). We concluded that the obese phenotype was an autosomal recessive monogenetic trait.

A candidate gene approach was employed to identify the *fatt* mutation, with the leptin and leptin receptor genes sequenced in the first instance. No mutations were observed in the leptin gene; however, in the LR coding gene a guanine to adenosine nucleotide substitution (1,279 G > A) was observed that segregated precisely with the obese phenotype. This 1,279 G > A base substitution affected the most 3′ protein-coding nucleotide in exon 7 (encoding the IGD in the receptor), adjacent to the GT splice donor of intron 7. RT-PCR analysis and sequencing on mRNA isolated from the hypothalamus showed that two new transcripts were generated in the obese mice. In the first transcript, exon 7 had been excised precisely from the transcript, juxtaposing exons 6 and 8. This splice variant (minus exon 7) will hereafter be referred to as LR-FATT1. The second RT-PCR product retained exon 7, but also the entire 152 bp sequence of intron 7. This alternatively spliced form of the LR transcript (plus intron 7) will hereafter be referred to as LR-FATT2 (Fig. 1b). The predicted protein translation products of these alternate transcripts are indicated in Fig. 1c.

### In vitro properties of the LR variant lacking the IGD

**Fig. 2:**
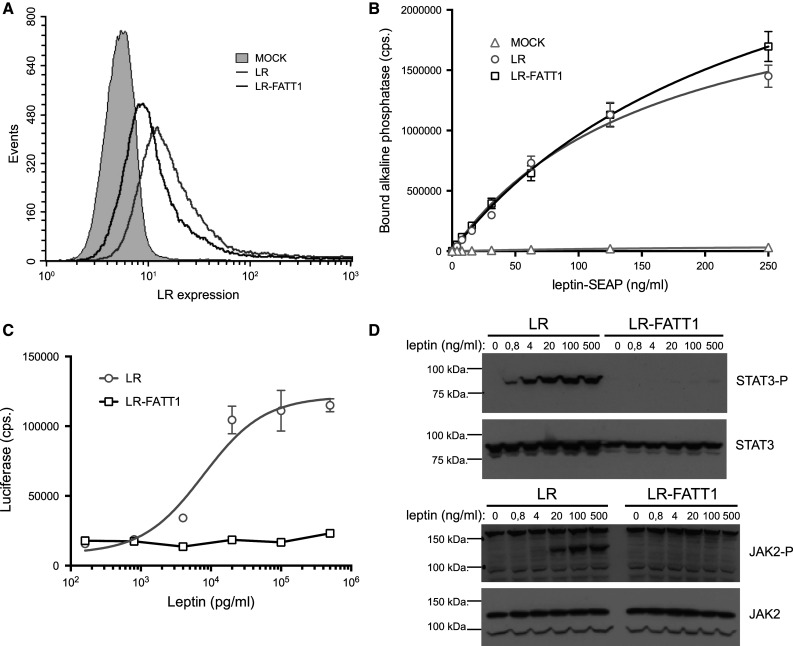
Expression, binding and signalling properties of LR-FATT1. **a** Hek293T cells were transiently transfected with plasmids encoding the full-length LR, LR-FATT1 or empty vector (MOCK). LR expression was monitored with FACS using mLR-specific antibodies. **b** LR, LR-FATT1 or MOCK transfected Hek293T cells were incubated with a serial dilution leptin-SEAP containing supernatants as indicated. Cells were washed and bound alkaline phosphatase activities measured. Mean values of quadruplicate measurements (±STDEV) are plotted. **c** Hek293T cells transfected with receptors and the STAT3-responsive rPAP1-luciferase reporter, were stimulated overnight with a serial dilution of leptin. Data points are the mean (±STDEV) of triplicate luciferase measurements. **d** Similar transfections were performed to check for phosphorylation of JAK2 and STAT3. Serum-starved cells were stimulated with indicated concentrations of leptin for 10 min or were left unstimulated. Lysates were blotted onto a nitrocellulose membrane and analysed using phospho-specific antibodies. Total amounts of JAK2 and STAT3 are also shown. All datasets are representative for three independent transfection experiments

FACS analysis was used to demonstrate that LR-FATT1 is expressed on the membrane upon transfection (Fig. 2a). Leptin-binding properties of wild-type LR and LR-FATT1 were compared using a leptin-SEAP (secreted alkaline phosphatase) binding assay on transiently transfected Hek293T cells. As shown in Fig. 2b, no differences were observed, thus ruling out effects of deletion of the IGD on ligand binding and cell-surface exposure. However, LR-FATT1 was completely defective in generating a STAT3-dependent signal as measured using a STAT3 responsive luciferase reporter construct (Fig. 2c). Western blot analysis of JAK2 and STAT3 phosphorylation demonstrated that the LR deletion variant was unable (up to leptin concentrations of 500 μg/ml) to stimulate JAK2 kinase activity and hence to initiate downstream STAT3 signalling (Fig. 2d).

### Phenotypic analysis of the *fatt/fatt* mice

Phenotypic characterization of the obese animals was conducted in 10- to 11-week-old homozygous obese mice (*fatt/fatt*) and compared to age-matched wild-type and heterozygous (*fatt/*+) mice. Briefly, *fatt/fatt* mice showed significant increases in body weight, the weight of fat pads and increased levels insulin (over tenfold compared to wild type), blood glucose and leptin (Table 1). The *fatt/fatt* mice had a longer body than wild-type and *fatt/*+ mice (males 13 %, females 20 % longer compared to wild type). *Fatt/fatt* mice of both sexes were infertile. Upon inspection, the reproductive tract of the female appeared poorly developed and *fatt/fatt* testes were small compared to non-obese males (Supplementary Fig. S1).

**Table 1:** Phenotype data for wild-type (+/+), heterozygous (fatt/+) and obese (fatt/fatt) FATT mice at 10–11 weeks of age

Sex	Males			Females		
Genotype	+/+	fatt/+	fatt/fatt	+/+	fatt/+	fatt/fatt
*N*	5	8	8	6	7	4
Body weight (g)	23.23 ± 2.68	23.39 ± 3.08	46.28 ± 3.86**^##^	16.63 ± 1.07	18.60 ± 2.16	38.92 ± 2.80**^##^
Body length (cm)	8.23 ± 0.74	8.28 ± 0.33	9.27 ± 0.27**^##^	7.28 ± 0.18	7.41 ± 0.59	8.70 ± 0.43**^##^
Femur length (mm)	15.00 ± 0.71	14.94 ± 0.62	14.42 ± 0.20	13.75 ± 0.69	13.86 ± 0.85	13.50 ± 0.50
Femur (mg)	35.93 ± 7.14	34.95 ± 6.00	36.45 ± 1.83	27.57 ± 5.64	27.63 ± 3.85	29.24 ± 2.83
Liver (mg)	905.50 ± 158.74	905.80 ± 123.58	1,551.70 ± 251.42**^##^	589.30 ± 55.61	694.60 ± 83.44*	1,068.80 ± 96.52**^##^
Liver (mg/body weight)	38.80 ± 2.95	39.10 ± 5.97	33.60 ± 5.81	35.60 ± 4.85	37.4 ± 2.55	27.70 ± 3.89**^##^
Gonadal WAT (% of body weight)	1.47 ± 0.10	1.86 ± 0.41	5.83 ± 0.77**^##^	2.04 ± 0.68	3.46 ± 1.19*	11.56 ± 0.53**^##^
Retroperitoneal WAT (% of body weight)	0.28 ± 0.06	0.34 ± 0.08	2.38 ± 0.25**^##G^	0.25 ± 0.07	0.42 ± 0.16	1.66 ± 0.31**^##G^
Subcutaneous WAT (% of body weight)	0.99 ± 0.31	1.09 ± 0.14	4.80 ± 0.64**^##G^	1.7 ± 0.52	2.24 ± 0.86	5.43 ± 0.28**^##^
Visceral WAT (% of body weight)	2.10 ± 0.3	2.01 ± 0.38	3.85 ± 0.72**^##^	1.96 ± 0.39	2.26 ± 0.25	3.42 ± 0.33**^##^
BAT(% of body weight)	0.36 ± 0.08	0.35 ± 0.05	0.74 ± 0.25**^##^	0.34 ± 0.09	0.40 ± 0.050	0.75 ± 0.16**^##^
Glucose (mM)	5.25 ± 1.66	5.79 ± 1.03	12.50 ± 3.40**^##G^	4.03 ± 0.97	4.89 ± 0.60	4.72 ± 1.18
Insulin (ng/ml)	0.79 ± 0.23	0.71 ± 0.47	73.10 ± 24.95**^##G^	0.31 ± 0.21	0.58 ± 0.31	26.91 ± 12.71**^##G^
Leptin (ng/ml)	7.10 ± 5.28	7.92 ± 6.51	116.36 ± 29.72**^##^	5.82 ± 3.60	7.98 ± 3.71	103.65 ± 25.51**^##G^
Age (days)	74.50 ± 3.0	75.80 ± 3.74	75.83 ± 2.86	72.17 ± 1.60	73.14 ± 3.24	73.00 ± 4.00

Results are displayed as average ± standard deviation

*WAT* white adipose tissue, *BAT* brown adipose tissue

Tukey’s HSD test used in all analysis unless noted, Games–Howell test (G): significance is denoted as * *P* < 0.05 versus +/+, ** *P* < 0.001 versus +/+, ^#^ *P* < 0.05 versus fatt/+, ^##^ *P* < 0.0.001 versus fatt/+. Differences are not significant unless noted

**Fig. 3:**
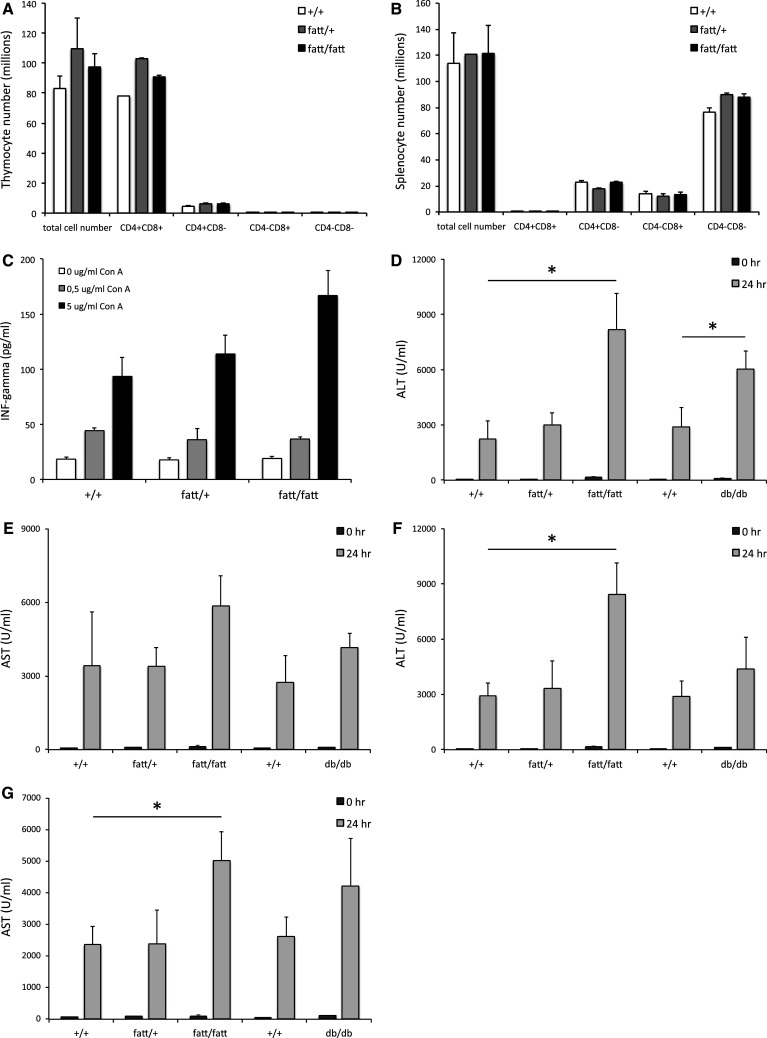
FATT mutation does not affect cellularity of the thymus and spleen and aggravates Con A-induced hepatitis. Thymic (**a**) and splenic (**b**) lymphocytes were isolated from 9- to 10-week-old wild-type (+*/*+), heterozygote (*fatt/*+) and obese (*fatt/fatt*) mice. Cells were counted and stained for CD4 and CD8 expression using FACS. *Bars* represent averages ± SEM. **c** Splenocytes were seeded in 96-well plates and stimulated with indicated amounts of Con A for 96 h. IFN-γ levels were determined in a specific ELISA. *Bars* represent averages of 4 mice; quadruplicate values per mouse and error bars the SEM. **d**–**g** Hepatitis in +*/*+, *fatt/*+ , *fatt/fatt*, or LR deficient *db/db* and +*/*+ littermates mice was induced by intravenous injection of Con A. Animals were treated either according to their weight (10 mg/kg; **d** and **e**) or with a fixed amount Con A (400 μg; **f** and **g**). Blood was collected at 0 and 24 h and serum ALT (**d**, **f**) and AST (**e**, **g**) levels are plotted as averages (*n* = 10) ±SEM. Data are representative for three or more experiments. Statistics: *P < 0.05; Mann–Whitney *U* test

Intriguingly, the thymus was increased in size and weight in 10-week-old *fatt/fatt* mice and the spleen appeared to be normal compared to age-matched wild-type controls (Table 2). Furthermore, the total number of thymocytes and splenocytes were comparable in wild-type, heterozygous and obese mice (Fig. 3a, b). We did not observe differences in the relative percentages in CD4 and CD8 double positive, CD4^+^, CD8^+^ and double negative populations in both thymus and spleen (Fig. 3a, b). Finally, splenocytes derived from *fatt/fatt* mice produced interferon gamma (INF-γ) in response to concanavalin A (Con A) comparable to cells from wild-type or heterozygous animals (Fig. 3c). Taken together, *fatt/fatt* mice represent the first case of LR deficiency with an intact immune compartment.

**Table 2:** Spleen and thymus characteristics wild-type (+/+), heterozygous (fatt/+) and obese (fatt/fatt) mice $

Sex	Males			Females		
Genotype	+/+	fatt/+	fatt/fatt	+/+	fatt/+	fatt/fatt
*N*	5	8	8	6	7	4
Spleen (mg)	132.13 ± 26.98	152.5 ± 33.91	149.20 ± 57.13	99.77 ± 17.46	113.51 ± 22.26	125.72 ± 43.77
Spleen (mg/body weight)	5.73 ± 1.25	6.54 ± 1.37	3.26 ± 1.37*^##^	6.05 ± 1.24	6.20 ± 1.53	3.26 ± 1.20**^##^
Thymus (mg)	46.30 ± 9.8	38.18 ± 14.38	67.12 ± 18.43^##^	42.60 ± 4.8	42.94 ± 8.92	68.04 ± 17.27**^##^
Thymus (mg/body weight)	2.03 ± 0.37	1.62 ± 0.76	1.37 ± 0.43	2.45 ± 0.33	2.15 ± 0.27	1.63 ± 0.52**^#G^
Age (days)	74.50 ± 3.0	75.80 ± 3.74	75.83 ± 2.86	72.17 ± 1.60	73.14 ± 3.24	73.00 ± 4.00

Results are displayed as average ± standard deviation

*WAT* white adipose tissue, *BAT* brown adipose tissue

Tukey’s HSD test used in all analysis unless noted, Games–Howell test (G): significance is denoted as * *P* < 0.05 versus +/+, ** *P* < 0.001 versus +/+, ^#^ *P* < 0.05 versus fatt/+, ^##^ *P* < 0.001 versus fatt/+. Differences are not significant unless noted

### LR deficiency does not suppress Con A induced hepatitis

To assess the importance of a signalling competent LR in immune responses in vivo, we compared obese *fatt/fatt* and *db/db* mice with wild-type and heterozygous littermates in the Con A mouse model for autoimmune hepatitis. In two series of experiments, animals were either treated according to their weight (i.e. 10 g/kg Con A; Fig. 3d, e), or with a fixed dose (i.e. 400 μg Con A per mouse; Fig. 3f, g). Blood ALT and AST levels were measured 24 h after treatment. Neither the homozygous LR *fatt* nor the *db* mutation resulted in a protection against Con A induced hepatoxicity (Fig. 3d–f). Liver enzymes were even significantly increased in some set-ups. Wild-type and *fatt/*+ animals reacted comparably to Con A. Together, these data show that LR deficiency results in the aggravation of an experimentally induced autoimmune disease.

### Administration of a nanobody targeting the IGD results in weight gain and hyperinsulinaemia

**Fig. 4:**
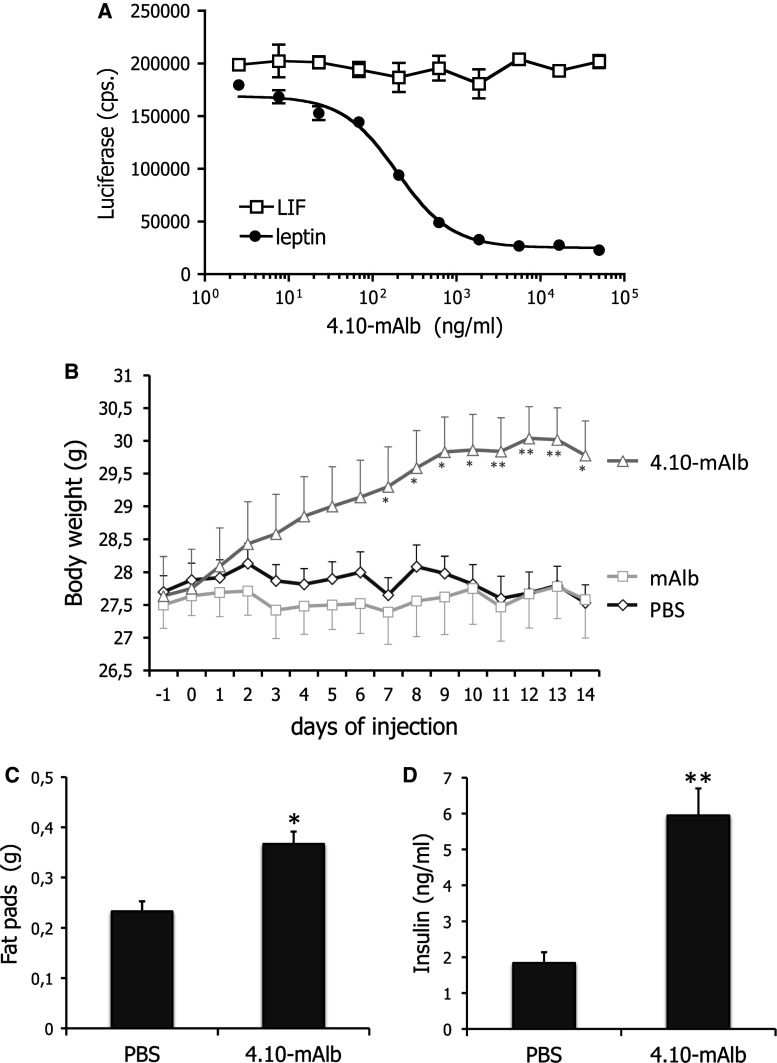
A nanobody targeting the LR IGD blocks leptin signalling in vitro and induces weight gain upon administration. **a** LR and rPAP1-luciferase reporter co-transfected cells were stimulated overnight with sub-optimal leptin or LIF concentrations in the presence of a serial dilution 4.10-mAlb. Luciferase activity was measured and plotted as average (±STDEV) of triplicate measurements. **b** Body weight changes of C57BL/6 mice injected daily with bispecific nanobody (40 μg/day/mouse; *n* = 10), the monovalent mAlb (also 40 μg/day/mouse; *n* = 10) or PBS (*n* = 8) were followed for 14 days. Data are expressed as averages ±SEM. Results are representative for at least four experiments. **c** Abdominal fat pad weights were measured in mice treated for 7 days with 4.10-mAlb (150 µg/mouse/day; *n* = 7) or PBS (*n* = 7) and plotted as averages (±SEM). **d** Serum insulin levels in PBS or 4.10-mAlb-treated mice (same as **c**) were measured as described in the “Materials and methods”. *Error bars* represent SEM. Data are representative for two experiments. Statistics: *P < 0.05, **P < 0.001, Mann–Whitney *U* test

Given the unexpected phenotype of *fatt/fatt* mice, we chose to test the neutralizing, IGD-specific 4.10 nanobody [28] in more detail in vivo. The nanobody was fused to a second nanobody that binds mouse serum albumin (4.10-mAlb) to prolong its half-life in circulation. This bispecific nanobody clearly blocked LR STAT3 dependent signalling without affecting the related LIF (leukaemia inhibitory factor) receptor activation (Fig. 4a).

4.10-mAlb, the monospecific nanobody mAlb or PBS was administered daily to 9- to 10-week-old C57BL/6 mice for 14 days and metabolic parameters were scored. Figure 4b clearly illustrates that 4.10-mAlb provoked a significant increase in body weight, accompanied by enlargement of abdominal fat pad mass and hyperinsulinaemia (Fig. 4c, d). Together, these data demonstrate that 4.10-mAlb is a potent inhibitor of leptin’s metabolic functions in vivo.

### Targeting the IGD does not suppress experimentally induced autoimmune diseases

To investigate whether the IGD is required for leptin’s immuno-modulatory functions, 4.10-mAlb was evaluated in three mouse models for autoimmune diseases: EAE, CIA and Con A-induced hepatitis.

**Fig. 5:**
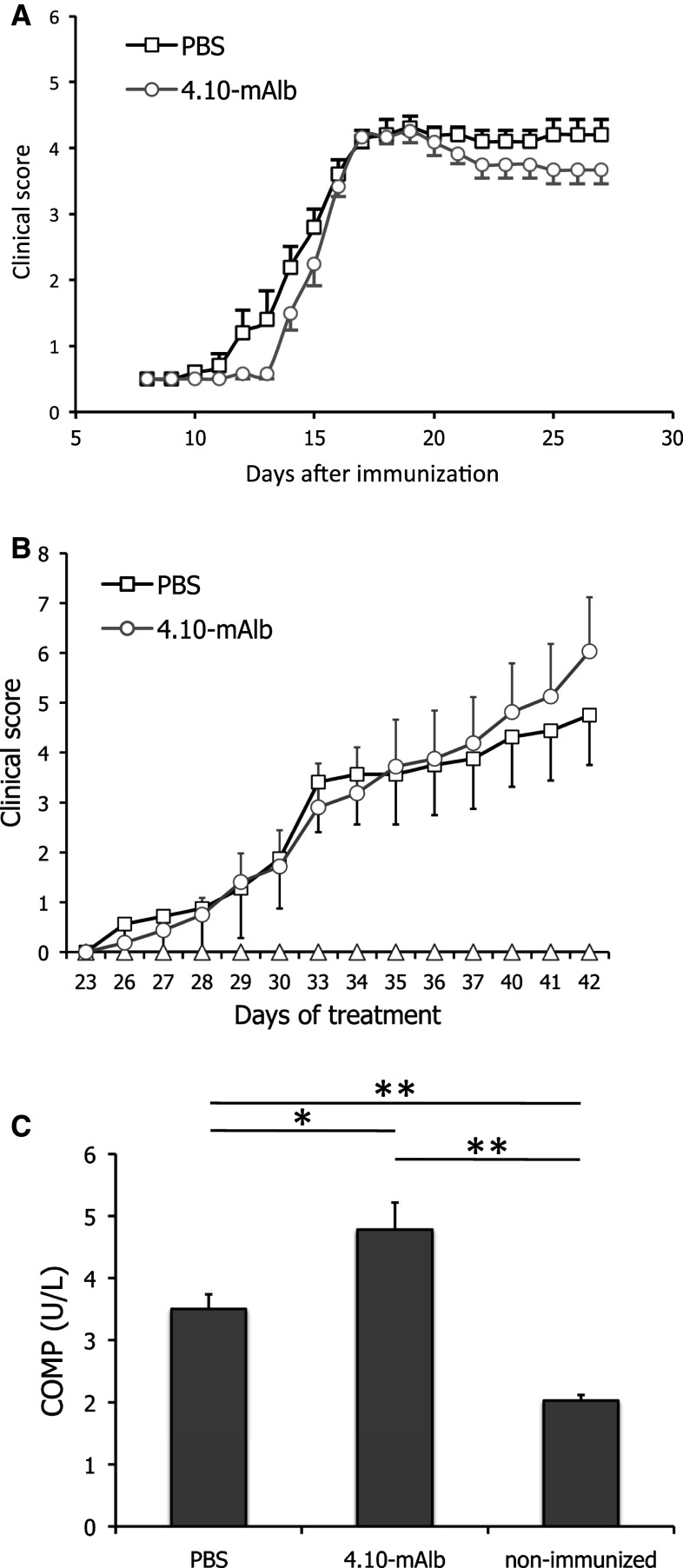
4.10-mAlb does not protect against experimental autoimmune encephalomyelitis (EAE) and collagen-induced arthritis (CIA). **a** EAE was induced in male C57BL/6 mice, and clinical symptoms were scored in PBS (*n* = 6) or 4.10-mAlb (100 μg/mouse/day; *n* = 7) treated mice after immunization with MOG_35–55_ peptide. Average clinical scores are plotted ± SEM. **b** Arthritis was induced in 9- to10-week-old DBA/1 mice with chicken type-II collagen on days 0 and 21. Starting at day 20, mice were daily injected with 4.10-mAlb (100 μg/mouse/day; *n* = 16) or PBS (*n* = 16). Average clinical scores are plotted ± SEM. **c** On day 42, blood was collected and COMP serum levels determined. *Bars* represent average (*n* = 16) ±SEM. Results are representative for two independent experiments. Statistics: *P < 0.05 and **P < 0.001 Mann–Whitney *U* test

In C57BL/6 J mice, EAE was provoked by administration of the MOG peptide (MOG_35–55_). Seven days after this immunization, animals were treated on a daily basis with 4.10-mAlb (100 μg/mouse/day) or PBS as a negative control and clinically scored as described in the “Materials and methods”. As shown in Fig. 5a, no statistically significant difference was observed between 4.10-mAlb- and PBS-treated mice as both followed a typical disease course and developed signs of severe paralysis, with an incidence of 100 % and reaching a mean maximal clinical score of 4.17 ± 0.067 (PBS) and 3.83 ± 0.21 (4.10-mAlb).

CIA was induced in DBA/1 mice by two (day 0 and 21) challenges with chicken type-II collagen. One group was treated semi-therapeutically (starting at day 20) with 4.10-mAlb, while PBS-injected mice served as a control. Body weight and clinical symptoms of arthritis were monitored daily until day 42 after the first immunization. Despite a clear effect on body weight (data not shown), no protection was observed in clinical arthritic scores (Fig. 5b). On the contrary, a tendency towards aggravation of the disease was seen in 4.10-mAlb-treated mice. The latter finding was consistent with a significant increase in serum levels of cartilage oligomeric matrix protein (COMP), a marker for cartilage damage (Fig. 5c).

**Fig. 6:**
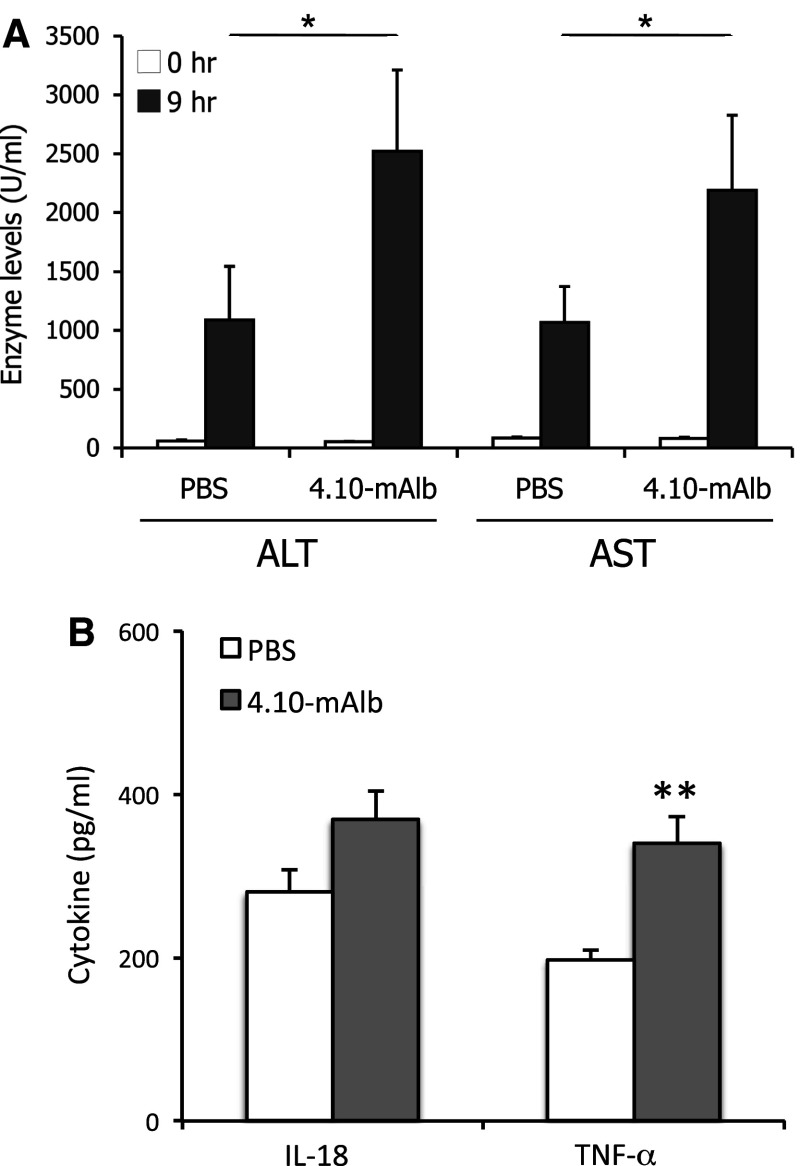
4.10-mAlb worsens the clinical outcome of ConA-induced hepatitis. **a** C57BL/6 mice were treated daily with 4.10-mAlb (150 μg/mouse/day; *n* = 15) or PBS (*n* = 15) for 1 week. On day 7, 300 μg ConA was injected intravenous and blood collected at 0 and 9 h. Serum ALT and AST are plotted as averages ± SEM. Results are representative of four independent experiments. **b** Serum IL-18 and TNF-α levels in these PBS and 4.10-mAlb-treated mice were measured and plotted as average ± SEM. Statistics: *P < 0.05 and **P < 0.001 Mann–Whitney *U* test

4.10-mAlb was also tested in the Con A mouse model for autoimmune hepatitis. 4.10-mAlb or PBS as a negative control was administered daily in C57BL/6 mice for 1 week. On day 7, Con A was injected intravenously and blood was collected at 0 and 9 h. Serum ALT and AST levels were significantly elevated in 4.10-mAlb-treated mice (Fig. 6a). The increase in hepatic injury coincided with a significant increase in tumour necrosis factor-alpha (TNF-α) and trend towards elevated serum interleukin-18 (IL-18) concentrations (Fig. 6b). Together with the EAE and CIA experiments, these results clearly illustrate that 4.10-mAlb is unable to block leptin’s immune-modulatory functions in all three disease models.

### Targeting the IGD induces hyperleptinaemia and a concomitantly increased T_H_1 profile

**Fig. 7:**
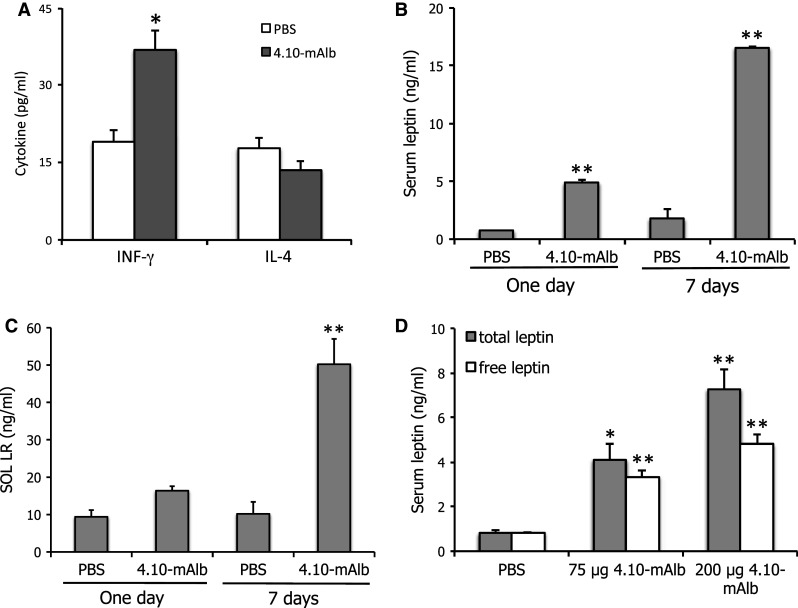
Nanobody-driven stabilization of soluble LR results in hyperleptinemia. **a** C57BL/6 mice were treated for 7 days’ treatment with 4.10-mAlb (150 μg/mouse/day) or PBS. Spleens in these conditioned mice were dissected and cells cultured. Cells were stimulated with Con A for 96 h, and IFN-γ and IL-4 levels were determined in specific ELISAs. *Bars* represent averages of 7 mice; quadruplicate values per mouse and error bars the SEM. **a**, **b** Mice were treated with 4.10-mAlb (200 μg/mouse/day) or PBS for 1 day or 1 week. Serum leptin (**a**) and SOL LR (**b**) were measured and plotted as averages (*n* = 10) ±SEM. **c** Mice were injected with 75 and 200 μg 4.10-mAlb or PBS. 24 h later, total and free leptin levels were determined as described in the “Materials and methods”. *Bars* represent averages (*n* = 10), *error bars* the SEM. Results are representative for three experiments. Statistics: *P < 0.05 and **P < 0.001 Mann–Whitney *U* test

Since leptin can modulate T_H_ responses [29], we isolated and cultured splenocytes from 4.10-mAlb- or PBS-treated mice and measured interferon-gamma (IFN-γ; a typical T_H_1 cytokine) and IL-4 (a typical T_H_2 cytokine) levels 96 h after stimulation with Con A (Fig. 7a). A significant increase in T_H_1 cytokine production was seen in 4.10-mAlb-treated mice, in line with the worsening of arthritis and hepatitis parameters. The effect on T_H_2 cytokine secretion was only marginal. To understand the paradoxical finding that a leptin antagonist can provoke an enhanced T_H_1 response, we measured circulating leptin levels in 4.10-mAlb-treated animals. Data shown in Fig. 7b demonstrate that 1-day or 1-week treatment resulted in a seven and tenfold increase in serum leptin, respectively. Since no effect on fat composition (and associated enhanced leptin secretion) can be expected after 24 h, this strongly suggested a direct effect on circulating leptin. Since the soluble LR (SOL LR) is able to stabilize leptin in circulation, SOL LR serum levels were measured after 1- or 7-day treatments. Data in Fig. 7c show an increase in both cases, thereby providing an explanation for an enhanced leptin serum half-life. To rule out that accumulation of SOL LR decreased the bioavailability of free leptin, we measured total and free leptin levels in a plate-binding assay using, respectively, an anti-leptin Ab or the LR ectodomain (see “Materials and methods” for details). Results in Fig. 7d illustrate that 4.10-mAlb treatment resulted in a clear increase in both total and free leptin levels in a dose-dependent manner. This increase in biologically active leptin levels provides an explanation for the aggravation upon 4.10-mAlb treatment of the hepatic damage in the Con A model.

## Discussion

In this study we describe the characterization of a new obese *fatt/fatt* mouse strain. The phenotype is caused by a single base mutation close to the border of exon 7 in the LR coding gene. This results in two new splice variants: one lacking exactly the IGD encoding exon 7 (in all LR isoforms) and one including intron 7 (Fig. 1b). Both resulting receptors LR-FATT1 (a membrane anchored form lacking the IGD) and LR-FATT2 (a soluble variant) are signalling deficient (Fig. 2c).

Like *ob/ob* (leptin deficient) and *db/db* (deficient in the LR long form) mice, *fatt/fatt* mice are not only massively obese, but also hyperinsulinemic, hyperglycemic (although more pronounced in male animals) and hyperleptinemic (Table 1) and infertile. The poor development of the female reproductive tract and the smaller testes in male *fatt/fatt* mice explains the infertility of the animals (Supplementary Fig. 1). In agreement with observations in *ob/ob* [30] and *db/*db [31] animals, *fatt/fatt* mice have an increased vertebral length, supporting the role of leptin in bone formation. In strong contrast to *ob/ob* mice [32], *db/db* mice [33–35] or even *fa/fa* rats [36], the size and cellularity of the spleen and thymus of *fatt/fatt* mice do not seem to be different (or is even significantly bigger in the case of the thymus) from heterozygous or wild-type animals (Table 2; Fig. 3). The relative percentages of CD4 and CD8 double positive CD4^+^CD8^+^, CD4^+^CD8^−^, CD4^−^CD8^+^ and double negative CD4^−^CD8^−^ thymocytes were similar in *fatt/fatt*, *fatt/*+ and wild-type animals (Fig. 3a, b), which was also observed in *db/db* mice [34]. Leptin deficiency, however, results in a marked decrease in double positive cells, while single positive and especially double negative cell numbers were significantly increased [32]. Finally, the homozygous *fatt* mutation did not result in altered INF-γ secretion by splenocytes (Fig. 3c).

To the best of our knowledge, this is the first report of a LR deficiency model in which the immune compartment appears to be maintained. To further investigate this, *fatt/fatt* mice were compared to wild-type and heterozygous littermates in the Con A-induced hepatitis model. Surprisingly, enzyme ALT and AST levels were significantly increased upon treatment with fixed doses of Con A or according to the animal’s weight (Fig. 3d–g). Also, we did not observe a protection of *db/db* mice in this mouse model (Fig. 3). The in vivo effects of the IGD-specific 4.10 neutralizing nanobody are completely in line with the observations in *fatt/fatt* mice. Treatment of mice with the bispecific 4.10-mAlb nanobody induces a significant increase in body weight and associated hyperinsulinaemia (Fig. 4), but failed to improve the clinical outcome of the CIA, EAE and ConA models for autoimmune diseases (Figs. 5, 6).

In our Con A experiments, LR deficiency (*fatt* or *db* mutation) or LR antagonism (4.10-mAlb) not only did not result in protection, but even aggravated the disease severity in some experimental set-ups. This may be explained by the substantial increase in circulating leptin in *fatt/fatt*, *db/db* or nanobody-treated animals. In the case of nanobody treatment, this increase is likely not only due to the increase in body fat but also a consequence of 4.10-mAlb-mediated stabilization of the SOL LR (Fig. 7). This bispecific nanobody is designed to tether 4.10 to albumin and thus will capture the SOL LR, thereby preventing its removal from circulation. Since 4.10 does not interfere with leptin binding, this may enhance leptin’s serum half-life. Indeed, not only the total but also the free leptin levels appear to be increased since the majority of leptin in serum is still able to bind the receptor in a plate binding assay (Fig. 7d). This mechanism of induced hyperleptinaemia is reminiscent of that found in Zucker Diabetic Fatty (ZDF) rats and liver-specific insulin receptor knock-out (LIRKO) mice, or as a consequence of over-expression of the SOL LR [37, 38]. This SOL LR stabilizing effect might also help to explain why the increase in body weight upon 4.10-mAlb treatment is only modest (less than 10 % of the initial body weight).

Our results appear contradictory to previous studies in *db/db* mice and indicate that leptin and LRlo deficiency affect immunity differently. *Ob/ob* mice are protected from immune-mediated inflammation in various disease models, such as experimental colitis [39], EAE [12, 13], Con A-induced hepatitis [14–16], or antigen-induced arthritis (AIA) [11]. *Db/db* mice develop a milder form of AIA [11], dextran sulphate sodium (DSS) or Con A-induced hepatitis [40]. To exclude the possibility that the experimental set-up causes our contradictory results, we treated animals either based on their weight, or with a fixed amount of Con A. In both cases, *fatt/fatt* and *db/db* mice reacted more to Con A than wild-type or heterozygous animals.

One could argue that leptin’s immune signalling in *db/db* mice is mediated, at least in part, by the LR short isoforms. Expression and functionality of these variants are not affected by the *db* mutation [41]. Although these short receptors lack cytoplasmic tyrosine residues and the so-called box 2 motif necessary for JAK2 activation [42, 43], they appear capable of signalling in certain experimental set-ups [44, 45]. This is, however, unlikely since the *fatt* mutation also results in the deletion of the IGD in these variants and since the short forms should also be sensitive to neutralization by the IGD-specific nanobody.

In line with our results, Palmer and colleagues were the first to postulate that the effects of LRlo deficiency on the immune system might be indirect and mediated by changes in the environment [34]. They showed that bone marrow cell transplants from *db/db* or wild-type mice to wild-type recipients resulted in similar size and cellularity of the thymus, as well as cellular and humoral immune responses. In contrast, wild-type to *db/db* grafts greatly affected the thymus fate. Also, macrophage infiltration of adipose tissue in a peritoneal dialysis mouse model was increased in *db/db* mice compared to wild-type littermates [46]. The authors suggested that this is mainly caused by the associated hyperleptinemia and that signalling occurs via the LR short form. Finally, a neutralizing (both binding of leptin and signalling) nanobody directed against the CRH2 domain in the LR also worsened the clinical outcome of Con A-induced hepatitis [47]. The authors suggest that leptin protects against T cell-mediated hepatitis via modulation of invariant natural killer (iNKT).

Differences in leptin versus LR deficiency were also observed in humans. In 2007, Farooqi and colleagues sequenced the *lr* gene in 300 subjects with hyperphagia and severe early-onset obesity [48]. This has led to the identification of five nonsense and four missense mutations that resulted in the ablation of leptin signalling. Several phenotypic features seen in subjects with LR deficiency are not as severe as those in subjects with leptin deficiency [49].

Three studies in patients illustrate that mutations in the leptin or LR genes do not affect metabolic and peripheral functions in the same way. A homozygous transition in the leptin-coding gene resulting in a L72S replacement in the leptin protein was identified in a 14-year-old child of non-obese Austrian parents. The mutation does not affect expression, but interferes with the secretion of the hormone. The child showed signs of a hypogonadotropic hypogonadism, but only mild obesity and a normal T cell responsiveness [50]. Nizard and colleagues reported the pregnancy of a morbidly obese patient with a rare homozygous LR mutation [51]. The child’s growth and development have been normal. More recently, in collaboration with Rogaev and co-workers, we characterised a genetic defect with a high incidence of early-onset morbid obesity, but with limited effects on immunity and fertility (Moliaka et al. in preparation).

In conclusion, we here provide genetic and biochemical evidence that LR deficiency (*fatt/fatt* and *db/db* mice) or LR antagonism (4.10-mAlb) has profound metabolic effects, but does not interfere with immune responses. Our study warrants caution for the use of LR-based antagonists (antibodies or nanobodies) in autoimmune diseases and certain cancers. However, the possibility to uncouple leptin’s metabolic and immune functions opens the potential for the design of selective leptin or LR antagonists (reviewed in [52]) that interfere with leptin’s peripheral functions without affecting central weight regulation.

### Electronic supplementary material

Below is the link to the electronic supplementary material.

Supplementary material 1 (PDF 195 kb)

## Abbreviations

ALT: Alanine aminotransferase
AST: Aspartate aminotransferase
CIA: Collagen induced arthritis
COMP: Cartilage oligomeric matrix protein
CRH: Cytokine receptor homology
EAE: Autoimmune encephalomyelitis
FN III: Fibronectin type III
IGD: Immunoglobulin-like domain
IL: Interleukin
INF-γ: Interferon gamma
JAK: Janus kinase
LR: Leptin receptor
SEAP: Secreted alkaline phosphatase
STAT: Signal transducer and activator of transcription
TNF-α: Tumour necrosis factor alpha
T_Reg_: Regulatory T cell
