# Supplementary material for: Leptin’s metabolic and immune functions can be uncoupled at the ligand/receptor interaction level
Source: Cell Mol Life Sci. 2014 Aug 7;72(3):629–44. doi: 10.1007/s00018-014-1697-x (PMC4293488; doi:10.1007/s00018-014-1697-x)
Supplement: Supplementary file 1 — Supplementary material 1 (PDF 195 kb) [file 18_2014_1697_MOESM1_ESM.pdf]

A

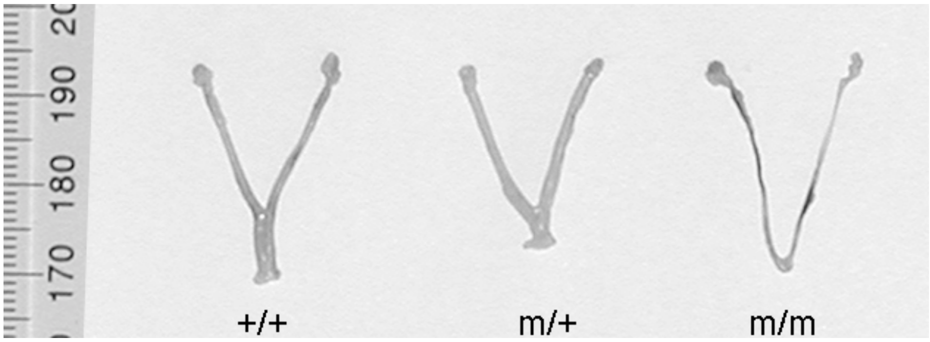

B

	Normal	Obese
**N**	4 (+/+ = 1, fatt/+ = 3)	3 (fatt/fatt)
**Testicle weight (mg)**	206.20 ± 23.95	168.81 ± 6.24

Supplementary figure 1: Reproductive organs of FATT mice.
(A) Reproductive tracts of female wildtype (+/+), heterozygous (fatt/+) and obese (fatt/fatt) mice. **(B)** Weights of the right testicle of male mice.
